# Spatio-Temporal Distribution and Influencing Factors of Human and Veterinary Pharmaceuticals in the Tributary Surface Waters of the Han River Watershed, South Korea

**DOI:** 10.3390/ijerph18157969

**Published:** 2021-07-28

**Authors:** Jong Kwon Im, Sang Hun Kim, Young Seuk Kim, Soon Ju Yu

**Affiliations:** Han River Environment Research Center, National Institute of Environmental Research, 42, Dumulmeori-gil 68beon-gil, Yangseo-myeon, Yangpyeong-gun, Gyeonggi-do 12585, Korea; haemy@korea.kr (S.H.K.); kys0522@korea.kr (Y.S.K.); ysu1221@korea.kr (S.J.Y.)

**Keywords:** emerging contaminants, downstream, wastewater treatment plants, non-prescription, livestock, population

## Abstract

Human and veterinary pharmaceuticals are being increasingly used for disease treatment; hence, their distribution and factors influencing them in the aquatic environment need to be investigated. This study observed the effect of human and animal populations, usage, purchasing criteria (prescription vs. non-prescription), and land use to identify the spatio-temporal distribution of eight pharmaceuticals at twenty-four sites of the tributaries of the Han River watershed. In rural areas, the mean concentration (detection frequency) of non-prescription pharmaceuticals (NPPs) was higher (lower) compared to that of prescription pharmaceuticals (PPs); in urban areas, a reverse trend was observed. Pharmaceutical concentrations in urban and rural areas were mainly affected by wastewater treatment plants (WWTPs) and non-point sources, respectively; concentrations were higher downstream (4.9 times) than upstream of the WWTPs. The concentration distribution (according to the target) was as follows: human–veterinary > human > veterinary. Correlation between total concentration and total usage of the pharmaceuticals was high, except for NPPs. Most livestock and land use (except cropland) were significantly positively correlated with pharmaceutical concentrations. Concentrations were mainly higher (1.5 times) during cold seasons than during warm seasons. The results of this study can assist policymakers in managing pharmaceutical pollutants while prioritizing emerging pollutants.

## 1. Introduction

Pharmaceuticals, including non-steroidal anti-inflammatory drugs (NSAIDs) and antibiotics, are used for treating human and animal diseases but are considered emerging pollutants, categorized into two classes [1,2]. Prescription pharmaceuticals (PPs) are drugs prescribed by a doctor for specialized medical treatment and non-prescription pharmaceuticals (NPPs) are drugs that do not require a prescription and can be purchased over-the-counter [3]. Antibiotics are used frequently in aquaculture, agriculture, and livestock farming as growth boosters [4]. While pharmaceutical drug use is increasing, owing to their benefits and the ongoing development of new pharmaceutical drugs [2,5], they are becoming environmentally ubiquitous in surface water, wastewater treatment facility effluent [6,7,8,9,10], groundwater [11,12], untreated sources of drinking water [13], and freshwater habitats in urban and agricultural areas [14].

Various factors influence the distribution patterns of pharmaceuticals in urban and rural surface water, including intake and usage patterns [15,16,17], population growth [18,19], population size of animals [20,21], land use [22,23], and physicochemical properties [24,25]. Particularly, the main reason for the existence of pharmaceuticals in the environment is that they cannot be completely treated in wastewater treatment facilities and very small quantities are removed by photolysis, adsorption, photodegradation, hydrolysis, electrolysis, and biodegradation in nature [26,27,28,29,30,31].

Unfortunately, large quantities of pharmaceuticals in surface water can endanger aquatic organisms such as bacteria, algae, invertebrate, and fish. Biotoxicity and susceptibility of organisms to the pharmaceuticals vary depending on the compounds and species [32,33,34,35,36]. Further, pharmaceutical compounds are transformed into various organic by-products as they decompose in treatment facilities and in the natural environment. The transformation by-products are toxic to aquatic organisms and can have higher toxicities than their parent compound [37,38], such as acridine, a transformation by-product of carbamazepine and oxcarbazepine, which has potential carcinogenic properties [39]. Likewise, O-desmethyl metoprolol (a by-product of metoprolol) [40], N-acetyl-p-benzoquinone imine and 1,4-benzoquinone (acetaminophen) [41], N-nitrosodimethylamine (Ranitidine) [42], and O-hydroxy atorvastatin and p-hydroxy atorvastatin (atorvastatin) [43] have higher toxicities than their parent compounds. [44,45,46,47]. Additionally, pharmaceutical concentrations in the environment are linked to pharmaceutical resistance, which has become a major environmental issue, arising from the improper use and disposal of pharmaceuticals [34,48,49].

While pharmaceuticals can affect water environments in various ways [50,51], their effects on humans are unclear. Furthermore, although pharmaceutical concentrations are increasing in wastewater treatment plants (WWTPs) and surface water, little information is available on the factors that influence the release of pharmaceuticals into the aquatic environment.

Studies have identified that WWTPs affect the distribution of pharmaceutical concentrations in surface water of the Han River watershed, yet, other factors affecting the distribution have not been identified [52,53,54,55,56]. Therefore, investigating the other factors affecting the distribution of various pharmaceuticals in surface waters is warranted.

In the present study, we discuss the results from a field study conducted from October 2015 to September 2016. The main objectives of this study were to: (1) investigate the overall distribution trend of eight pharmaceuticals; (2) compare PPs and NPPs at upstream and downstream areas of WWTPs; (3) identify the effects of human and animal populations, drug use (prescription and non-prescription), and land use on the distribution of the pharmaceuticals and the respective correlations; (4) describe the spatio-temporal variations in pharmaceutical concentrations and their possible pollutant sources in the tributaries of the Han River watershed, South Korea.

## 2. Materials and Methods

### 2.1. Chemicals and Materials

Eight pharmaceutical compounds (purity > 98%): naproxen (NPX), acetylsalicylic acid (ASA), carbamazepine (CBZ), trimethoprim (TMP), clarithromycin (CTM), sulfamethazine (SMZ), sulfamethoxazole (SMX), and sulfathiazole (STZ) were purchased from Sigma-Aldrich (St. Louis, MO, USA). The details of these pharmaceutical compounds are given in Table S1. The pharmaceutical compounds measured in this study were selected because of their high detection frequencies in previous studies [57]. Isotopically labeled internal standards (ISs), including tert-butylamine (TBL), sulfamethoxazole-d_4_ (SMZ-d_4_), and ibuprofen-d_3_ (IBP-d_3_), were purchased from Toronto Research Chemicals (Oakville, ON, Canada). HPLC grade solvents such as methanol and acetonitrile, and Na_2_-EDTA (analytical grade) were obtained from Sigma-Aldrich. Reagent water was prepared by Milli-Q water purification system (Millipore, Bedford, MA, USA). Individual stock solutions of each compound were prepared in methanol; standard mixtures were prepared by diluting the stock solution and stored in the dark at –20 °C. All glassware were baked at 250 °C overnight in the oven, and rinsed with methanol prior to use.

### 2.2. Study Sites and Sample Collection

**Figure 1:**
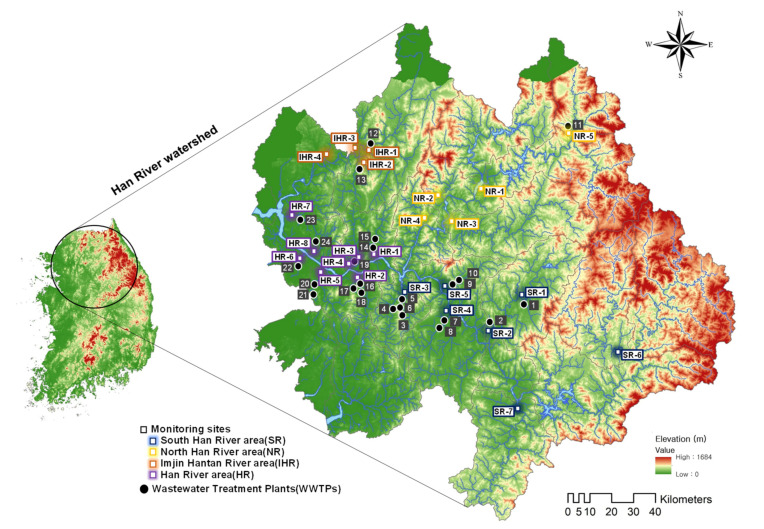
Map of the study area depicting the study sites in the Han River watershed. Sample sites and wastewater treatment plants (WWTPs) along the tributaries are denoted as squares and dots, respectively.

The Han River watershed is the largest in South Korea, consisting of four major rivers (Han, Nakdong, Geum, and Yeongsan rivers) and includes Seoul (average of 9,736,962 inhabitants in 2015–2016) and Gyeonggi Province (average of 13,265,377 inhabitants in 2015–16). Several tributaries flowing into the Han River are impacted by human activities in Seoul. In this study, human and livestock population and land use data were retrieved from the Korean Statistical Information Service (KOSIS) [58]. For this study, the watershed was divided into four areas (South Han River (SR), North Han River (NR), Imjin Hantan River (IHR), and Han River (HR)) to understand the characteristics of the representative tributaries, and 24 monitoring sites were selected (Figure 1).

Twenty-five WWTPs, except for those with a small treatment capacity (<1.0 m^3^/day), were identified; details are presented in Table S2. The study area is heavily influenced by a subtropical stormy climate: hot and humid in summer and cold and dry in autumn and winter. The average temperatures were 18.1 °C, 16.5 °C, 25.7 °C, and 25.3 °C in October 2015 and April, June, and September in 2016, respectively. Grab samples of surface water at 24 sites were collected on 14–16 April (spring), 18–19 June (summer), and 9–12 September 2016 (autumn). The 1000 mL samples were stored at 4 °C in a refrigerator before being treated and analyzed.

### 2.3. Sample Preparation and Analysis

Water samples (2 × 500 mL), filtered using 0.22 µm cellulose acetate membrane (Advantec, Toyo Roshi Kaisha, Ltd., Tokyo, Japan), were spiked with 25 µL isotopically labeled standard mix and 1000 µL of Na_2_-EDTA in water. Oasis HLB (500 mg–6 mL) solid phase extraction (SPE) cartridges were used to extract the compounds from the water samples. Preconditioning of cartridges was carried out in the following order: 4 mL water → 4 mL methanol → 4 mL water → 5 mL water (6.0 N hydrochloric acid, adjusted to pH 2). The sample was loaded at a rate of about 10 mL/min using SPE cartridges after conditioning. Cartridges were washed with 6 mL acidified water to extract any residual Na_2_-EDTA prior to drying under vacuum, for approximately 1 h. Analytes were eluted with 6 mL methanol and 12 mL of methanol/acetone (1/1, *v*/*v*). The extracts were evaporated to 0.5–1 mL with a gentle stream of high-purity N_2_.

The eight pharmaceuticals and ISs were quantified by ultra-performance liquid chromatography-tandem mass spectrometry (UPLC-MS/MS) coupled to Quattro Premier MS (Waters Acquity^TM^, Milford, MA, USA) equipped with an Agilent Eclipse plus C18 column (1.8 µm, 2.1 mm × 50 mm, Agilent, Palo Alto, CA, USA). The eluent flow rate was 0.25 mL/min with a gradient of eluent A (acetonitrile) and B (5 mM ammonium acetate). The gradient program was as follows: 15% A to 2 min, linear increase of B, A to 90% in 3 min and held for 2 min. The total run time was 15 min. A returned to 15% in 3 min and was stabilized for 5 min to finish the cycle. Injection volume of 10 µL was used for analysis and the column temperature was maintained at 40 °C. For MS detection, the instrument was operated in the positive electrospray ionization and multiple reactions monitoring (MRM) mode. The MS/MS parameters were optimized as follows: nebulizer pressure, 35 psi; gas flow, 8 L min^−1^; mass range, 100–400 amu; scan time, 300 ms; temperature, 350 °C. Other parameters of MS/MS and retention time are summarized in Table S3.

### 2.4. Quality Assurance and Quality Control (QA/QC)

QA/QC procedures were conducted to ensure the identification and quantification of the pharmaceuticals.

The pharmaceuticals’ method detection limit (MDL) and limit of quantitation (LOQ), classified as the concentrations corresponding to the signal-to-noise (S/N) ratios of 3 and 10, respectively, were 0.0008–0.013 µg/L and 0.0026–0.0413 µg/L, with spiked concentration between 0.005 µg/L and 0.03 µg/L. The pharmaceutical recovery and precision rate for the filtered surface water samples ranged from 91.2% to 113.7% and 1.8% to 15.2% for all analytes. The calibration curve was generated over a broad concentration range (0.2–1000 µg/L) to demonstrate high linearity (r^2^ > 0.98). Detailed information is given in Table 1.

### 2.5. Prediction of Sorption Tendency

The lipophilicity of a variety of pharmaceuticals is reduced when they are ionized at various pH values. K_ow_’s pH dependence can be expressed in D_ow_ as follows [59,60].

Dow = K_ow_/(1 + 10^pKa−pH^) for acidic compounds (1)

Dow = K_ow_/(1 + 10^pH−pKa^) for basic compounds (2)

Dow = K_ow_ for neutral compounds (3)

### 2.6. Statistical Analysis

Minitab 15 (Minitab Inc., State College, PA, USA) and Excel 2016 (Microsoft Co., Redmond, WA, USA) were used to conduct the statistical analysis. Pearson’s correlation analysis was conducted to identify sources using pharmaceutical concentrations, and one-way analysis of variance (ANOVA) and two-sample paired *t*-tests were used to compare pharmaceutical concentrations. Sigmaplot 12.0 (Systat Inc., Point Richmond, CA, USA) was used to generate graphics, and ArcGIS 9.2 (ESRI, Redlands, CA, USA) was used to produce a digital map of the Han River watershed.

## 3. Results and Discussion

### 3.1. Overall Trend of Pharmaceutical Distribution

**Figure 2:**
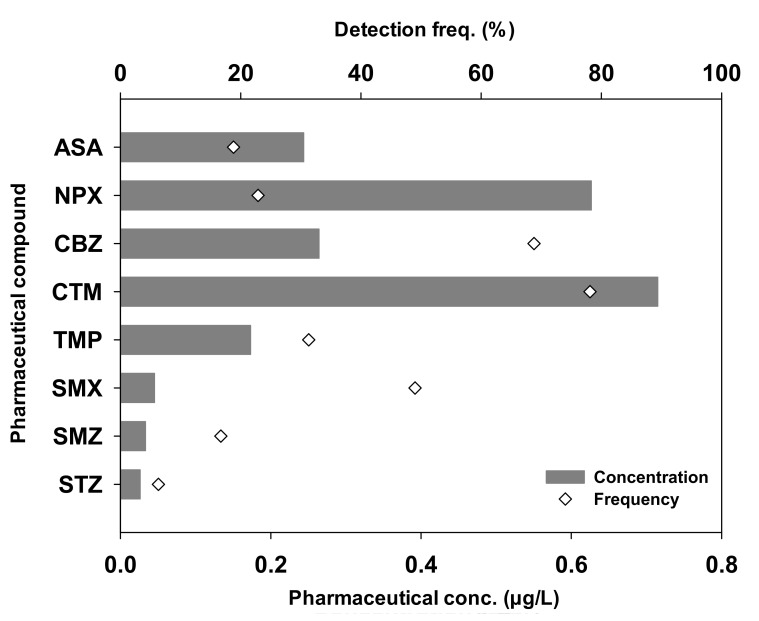
Concentration and detection frequency of eight pharmaceutical compounds measured in 24 water samples in the tributaries of the Han River watershed.

Concentrations and detection frequencies of the target pharmaceuticals in the tributaries of Han River watershed are shown in Figure 2. Each pharmaceutical was detected in all samples, with differing concentrations. CTM and CBZ were most frequently detected with detection frequencies of 78.1% and 68.8%, respectively. NPX, conversely, had a higher concentration than CBZ. Unlike CBZ, non-prescription drug NPX is widely used, and its relatively high concentrations in surface water are predicted. However, according to the findings of Zhang et. al. [61] about the removal mechanism of both pharmaceuticals, photodegradation and biodegradation played minor roles in CBZ removal, providing evidence of their resistance to natural degradation, while NPX showed high potential for both photodegradation and biodegradation. These results can also be found in other studies [16,62,63,64]. In addition, the effects of wastewater influent constituents may differ from site to site [65]. Therefore, NPX showed a low detection frequency despite its relatively high concentration. The detection frequencies of NPX, ASA, TMP, SMN, SMX, and STZ ranged from 6.3% to 49.0%. The mean concentrations of the pharmaceuticals varied significantly ranging from 0.0521 µg/L to 0.1822 µg/L. Among all the target compounds, CTM was detected at the maximum and total concentration of 0.1287 µg/L and 13.6648 µg/L, respectively. Various concentration levels of CTM with low body metabolic rates [48] and removal efficiency [66] have been widely reported in the surface waters in many countries [67,68,69,70], revealing its high consumption globally. Mean concentrations of other pharmaceuticals were in the following order: NPX > CBZ > ASA > TMP > SMX > SMN > STZ.

The distribution of concentration of the pharmaceutical compounds is related to their properties [25]. Although K_ow_ influences the sorption of different chemicals in WWTPs, pH may also influence sorption behavior; hence, D_ow_’s pH-dependent n-octanol water distribution ratio was estimated using the median pH 7 observed in this study [59] (Table S1). Generally, sorption is typically considered to be more prevalent for organic compounds that are highly hydrophobic (Log K_ow_ > 4; Log D_ow_ > 2.5) and have a large molecular weight (MW > 500 g/mol) [71,72]. However, since the pharmaceuticals in this study are not hydrophobic (Log K_ow_ < 4; Log D_ow_ < 2.5), removal by sorption would be negligible. Although pharmaceuticals are not recalcitrant pollutants in surface water (T ½ = 151.2–1440 h), the importance of continuous discharge should not be overlooked.

### 3.2. Distribution of Non-Prescription vs. Prescription Pharmaceuticals and Their Concentrations Upstream vs. Downstream

While most pharmaceuticals generally require a prescription, some are readily available at convenience stores without a prescription [73]. Therefore, comparing the mean concentration and detection frequency of PPs and NPPs, the NPPs had a mean concentration and detection frequency that were 1.8-times higher and 2.0-times lower than the PPs (Table 1). Additionally, the mean PP concentration was 4.3-times higher in urban areas, whereas NPPs were 1.4-times higher in rural areas. The detection frequency was 1.9- and 2.1-times higher for PPs in urban and rural areas, respectively. Specifically, the mean NPP concentration was relatively high in rural areas, whereas the detection frequency was high for PPs in urban areas. The mean concentration and the detection frequency of PPs were higher in urban areas than those in rural areas (Table 2).

**Figure 3:**
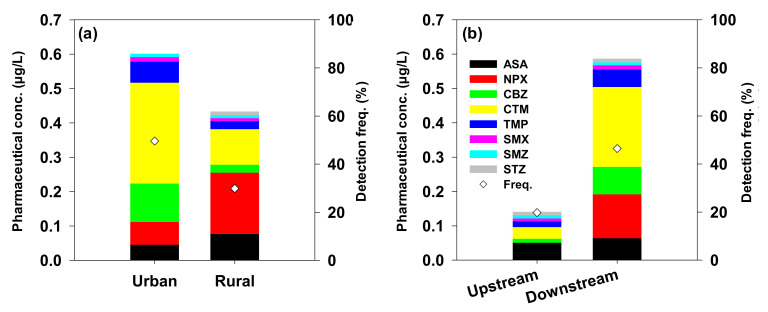
Concentration and detection frequency of the eight pharmaceuticals in (**a**) urban and rural regions and (**b**) upstream and downstream of the WWTPs.

The high detection frequency of the PPs in both urban and rural areas can be attributed to the continuous discharge from WWTPs (point source), whereas the relatively high mean concentration of NPPs in the rural areas can be attributed to the untreated discharge from public and private facilities such as parks, museums, camping sites, and arboretums, as well as to a floating population [13,56,74]. Additionally, previous studies state that the increased use and mobility of the NPPs might be responsible for their relatively higher presence in upstream areas compared to the PPs [11,75]. Regardless of their prescription state, the mean concentration and detection frequency of pharmaceuticals in urban areas were higher than in rural areas, as shown in Figure 3a, which could be attributed to the effect of point pollution sources (WWTPs).

The concentration of pharmaceuticals upstream and downstream of the WWTPs was examined. Of the twenty-four sites, nine and fifteen sites were present upstream and downstream, respectively. The total concentration and detection frequency downstream of the WWTPs was 4.9- and 2.4-times higher than those upstream, as shown in Figure 3b. This result can be attributed to the WWTPs as the primary source of pharmaceuticals [18]. Thus, if these facilities improve existing removal methods for pharmaceuticals, they could effectively reduce pharmaceutical discharge downstream. Other studies have also recorded higher pharmaceutical concentrations downstream compared to upstream sites [76,77]. Additionally, in various countries, namely, Mexico [13], Finland [78], the USA [7], China [79,80], and Japan [81], WWTPs were identified as the primary source of pharmaceutical compounds. Consequently, in this study, the distribution of PPs and NPPs in the Han River watershed surface water could be mainly attributed to point source pollution.

### 3.3. Effect of Human and Animal Populations, Usage, and Land Use on the Concentration and Distribution of Pharmaceuticals

**Figure 4:**
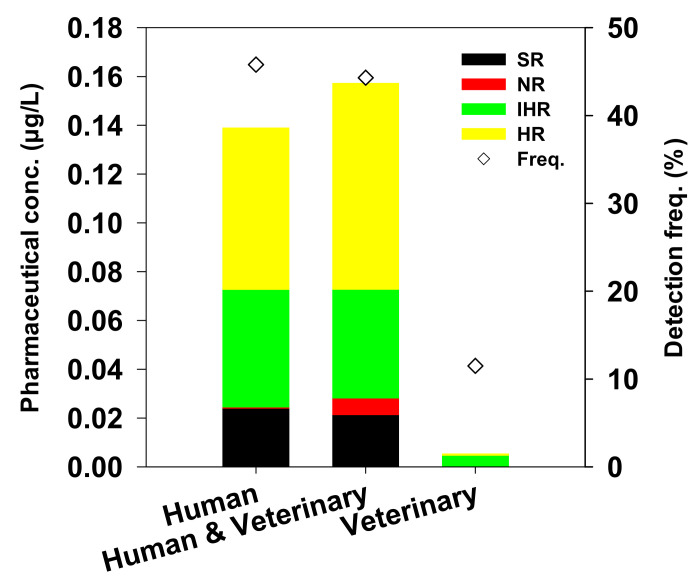
Comparison of concentration and detection frequency of pharmaceutical compounds in the three target categories of human, human and veterinary, and veterinary.

Pharmaceutical compounds selected in this study are divided into human, human and veterinary, and veterinary according to their intended use and detection concentration (Figure 4). Pharmaceuticals used in the human and veterinary, and human categories showed high mean concentrations and detection frequencies in urban areas with high population density, except for the detection frequencies of NPX (this could be attributed to the aforementioned physicochemical properties of NPX). It is only to be expected that the use and discharge of pharmaceuticals in urban areas will be high, owing to the large population in these areas. However, owing to well-managed modern WWTPs, the majority of the pharmaceutical compounds can be largely removed, and detected only in trace amounts relative to the amount of pharmaceutical compounds used in nearby rivers, as also reported in other studies [13,16,18].

By contrast, the relatively high proportion of rural areas and natural tourist attractions can cause the pharmaceuticals to be generated in the form of non-point pollutants, and their high concentrations were detected owing to the low removal efficiency of relatively old and small WWTPs (Table S2). SMZ, which is used only in the veterinary category, was relatively high in rural areas in terms of both mean concentration and detection frequency [82], while STZ was undetected in urban areas, where the population is a major source of pharmaceuticals [22].

Sun et al. [15] reported a high detection frequency of antibiotics in Chinese rivers, explored the relationship between the high production of four macrolides and the widespread use of three sulfonamides, and concluded that the high detection frequency of pharmaceuticals was related to the usage. Additionally, high amounts of nicotine and caffeine in Spain’s surface and tap water were attributed to the heavy consumptions of cigarette, coffee, and tea [83].

Concentration of pharmaceuticals in surface water have been reported to be a function of the population, livestock species and number, and land use types, as well as the population not connected to WWTPs as the potential pollutant sources; studies on correlation among these variables have been conducted in different countries [18,20,81]. However, similar studies on the Han River watershed in South Korea are lacking.

Table 2 summarizes the correlation between potential pollutant sources and pharmaceutical concentrations in urban and rural areas. The correlation between the total concentration of the pharmaceuticals and its total usage was insignificant. However, the r value was high for the correlation between the total concentration and total usage of the pharmaceuticals except for the NPPs ASA and NPX (r = 0.948). This is because ASA and NPX can be easily purchased over-the-counter; hence, these may have already been purchased in large quantities and stored as household pharmaceuticals, and their exact amount cannot be calculated or predicted [84,85]. Additionally, NPPs have a variety of discharge routes because they are primarily discharged in the form of non-point pollutants [16]. No correlation was observed between the total concentration and the population not connected to WWTPs, but the correlation was high with the population not connected to WWTPs except for urban areas. This implies that the total concentration of population not connected to WWTPs in rural areas and pharmaceutical concentrations are highly correlated, and several pharmaceuticals that were sourced from non-point pollution sources or improper treatment by WWTPs in rural areas were found to be present in high concentrations.

Table 3 shows the results of correlation analysis of the effect of livestock (dairy cattle, swine, and poultry) and land use (cropland, paddy land, forest land) on the total concentration of the pharmaceuticals detected. All livestock and land, except cropland, were found to be significantly correlated with the concentration of pharmaceuticals (*p* < 0.05). In cropland, herbicides and chemicals necessary for farming are mainly used rather than pharmaceuticals; hence, the proportion of pharmaceuticals utilized in the human and veterinary category is relatively very little [73]. Livestock population was found to be proportional to the concentration of pharmaceuticals observed in surface water by Osorio et al. [20]. In addition, Hanamoto et al. [81] concluded that cattle and swine urine influenced the concentration of pharmaceutical compounds.

### 3.4. Spatio-Temporal Variations and Possible Pollutant Sources

**Figure 5:**
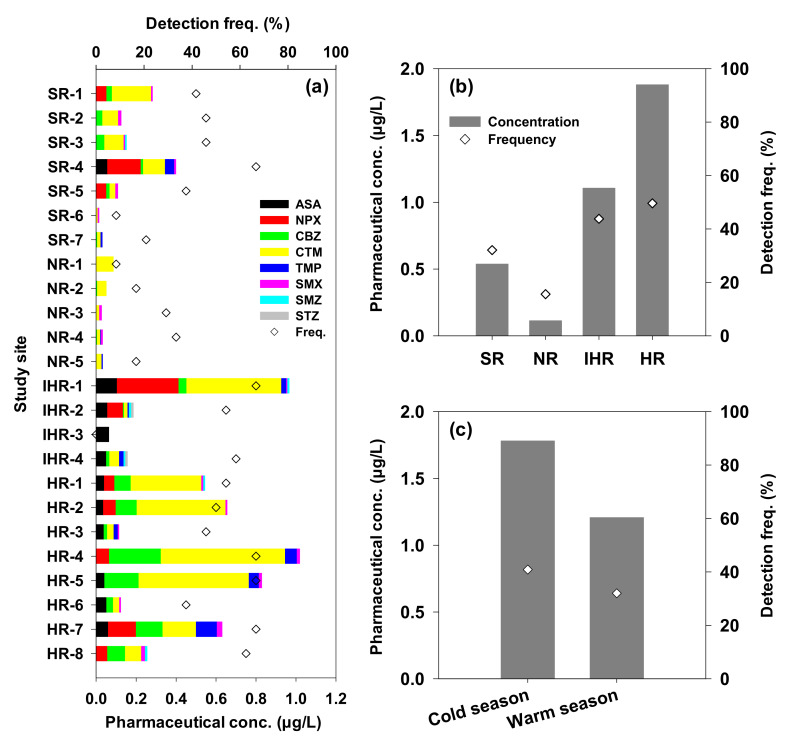
Concentration and detection frequency of the eight pharmaceuticals (**a**) at each of the 24 sites, (**b**) in the four areas, and (**c**) in the cold and warm seasons.

The concentration distribution of pharmaceutical compounds in independent rivers in the Han River watershed was analyzed (Figure 5a and Table S4). In the HR area with a relatively high population density and developed commercial facilities, the mean concentration and detection frequency were high, followed by IHR, SR, and NR (Figure 5b).

HR-4, where the highest pharmaceutical concentration was detected in the HR area, is located directly under the largest WWTP-19 in Seoul; the mean concentration was 10.1 times higher than that at HR-3 which is located upstream on the same river and is not affected by WWTPs. The pharmaceutical concentration in the HR area was found to be in the following order: HR-4 > -5 > -2 > -7 > -1 > -6 > -3 ranging from 0.0042 µg/L to 0.8661 µg/L with 12.5–75.0% detection frequency.

The second highest pharmaceutical concentration and detection frequency were in found in the IHR area. IHR-1 was detected having the second highest concentration among the 24 sites. However, unlike HR-4, this site does not have a WWTP located upstream, but a large recreational park is located nearby. This park is considered a non-point pollution source, unlike the WWTPs, which is a point source of pollution. Poor park management and careless waste discharge (such as that from toilet facilities) can lead to pharmaceutical compounds being present in high concentrations in the nearby areas. Other sites, IHR-2, -3, and -4, similarly detected low concentrations ranging from 0.0052 µg/L to 0.0859 µg/L.

The third highest concentration was detected in the SR area. SR-6 and -7 are not characterized by a WWTP upstream; they showed the lowest concentrations of pharmaceuticals while relatively high concentrations were detected at SR-4. Due to the mixed impact of non-point pollution sources such as livestock and farming, the Bok River, where SR-4 is located, receives pollutants from non-point sources such as livestock and farming [86]. Two WWTPs, WWTP-7 and -8, positioned upstream, are also considered to contribute towards high pharmaceutical concentrations at SR-4.

The NR area was characterized by the least amounts of pharmaceutical compounds among the areas studied; each site had very low levels of pollutants. The concentration of pharmaceutical compounds was different at each site in each river, and this is attributed to the capacity of WWTPs, the removal rate of pollutants by the WWTPs, and the content of organic matter in the WWTP effluent [87].

The water temperature was divided into cold (<20 °C, October 2015, April 2016) and warm (>20 °C, June and September 2016), corresponding to the seasons, and the concentration of pharmaceutical compounds in each season is shown in Figure 5c. Higher concentrations generally corresponded with the cold season, wherein the mean concentrations and detection frequencies of the selected pharmaceuticals were 1.3 and 1.5 times, respectively, higher than those in the warm season. The concentrations of all the pharmaceuticals in the cold season were higher than in the warm season. South Korea, located in a subtropical region, has cold and dry weather in winter and high temperatures and humidity caused by monsoons in summer, which probably has a dilution effect on the pharmaceutical concentrations. Higher usage of pharmaceuticals was found to occur in the cold season, which was evidenced by their high mass loads in surface water during this season [88,89,90,91,92]. However, hydrolysis, photodegradation, and biodegradation are typically more pronounced in the warm season [78], leading to faster attenuation of pharmaceuticals in the river [28]. In this study, seasonal variations appeared to be compound-dependent and may additionally be associated with either societal factors (e.g., production, consumption, and excretion) or environmental factors (e.g., solar irradiance, precipitation, and temperature). Furthermore, in the WWTPs in Korea (Table S2), a decrease in the activity of microorganisms is observed when the water temperature is low, resulting in relatively low pollutant degradation efficiency [7]. Therefore, it can be proposed that management should mainly focus on the downstream of the WWTPs in the HR area in the cold season.

### 3.5. Strengths and Limitations

This study has several strengths and limitations. First, to the best of our knowledge, this is the first study to identify the distribution characteristics of prescription and non-prescription pharmaceuticals in tributaries of the Han River. Second, we found that pharmaceuticals originated from human and animal populations and drug and land use. Third, we demonstrated high-quality analytical performance (i.e., high recovery, precision, and linearity) due to proper equipment maintenance during the analysis of pharmaceuticals in surface water samples. These results provide insight into the distribution of pharmaceutical substances in surface water based on environmental factors.

However, some limitations should be considered when interpreting our findings. First, a total of four water samples may not reflect the concentration of general pharmaceutical compounds representing the Han River watershed. Although sampling was performed at the same place and time, no data were collected for unusual environmental events, such as heavy rain and water structure construction. Second, confounding factors, such as regional pharmaceutical usage, an aging population, and major diseases may affect the results of this study. These factors could be the focus of future research.

Therefore, continued research and data collection will inform policymakers, securing the health of the aquatic ecosystem in surface water. Moreover, policymakers can use the comprehensive results of this study to determine the concentration and detection frequency of pharmaceuticals in the Han River watershed and consider the possibility of pharmaceutical discharge due to environmental factors.

## 4. Conclusions

In this study, eight pharmaceutical compounds of different therapeutic classes were investigated at twenty-four sites of the tributaries in the Han River watershed. The effects of various factors on pharmaceuticals distribution were investigated. Each of the studied pharmaceuticals were detected at least once in all of the samples. The mean concentrations of the pharmaceuticals showed the following order: CTM > NPX > CBZ > ASA > TMP > SMX > SMN > STZ with detection frequency ranging from 6.3% to 78.1%. In urban areas, the mean concentration and detection frequency of the PPs were higher than those of the NPPs, and in urban and rural areas, the pharmaceutical concentrations were significantly higher downstream of the WWTPs than upstream; this is mainly attributed to the WWTPs. Overall, human and human–veterinary pharmaceuticals were more abundant and were measured in higher concentrations compared to the veterinary pharmaceuticals. All livestock and land use (except cropland) were found to have a significant positive correlation with the concentration of pharmaceuticals (*p* < 0.05). Higher concentrations were mainly observed during the cold season, with the mean concentration being 1.3 times higher than that during the warm season. Spatial analysis revealed that the pharmaceutical concentrations in different areas were impacted by different sources. This study provides scientific information to policymakers for regulating the concentrations of pharmaceutical pollutants in natural aquatic environments, as well as identifying priority emerging pollutants.

## Figures and Tables

**Table 1:** Method detection limit (MDL) and limits of quantification (LOQ) for the target pharmaceuticals and their concentrations.

Classification	Compounds	Common Use	MDL (µg/L)	LOQ (µg/L)	Recovery (%)	Precision (%)	Linearity
NSAIDs	ASA ^(1)^	Human Veterinary	0.0108	0.0342	98.9	1.8	0.9993
NSAIDs	NPX ^(1)^	Human	0.0130	0.0413	96.9	15.2	0.9962
Antiepileptics	CBZ ^(2)^	Human	0.0013	0.0042	107.8	7.9	0.9998
Antibiotics	CTM ^(2)^	Human Veterinary	0.0028	0.0089	93.3	7.8	0.9844
Antibiotics	TMP ^(2)^	Human Veterinary	0.0008	0.0026	91.2	3.1	0.9991
Antibiotics	SMX ^(2)^	Human Veterinary	0.0013	0.0041	113.7	5.4	0.9833
Antibiotics	SMZ ^(2)^	Veterinary	0.0014	0.0043	108.3	3.5	0.9994
Antibiotics	STZ ^(2)^	Veterinary	0.0018	0.0056	113.1	4.9	0.9992

^(^^1)^ non-prescription pharmaceutical (NPP), ^(2)^ prescription pharmaceutical (PP).

**Table 2:** Concentration, detection frequency, and ratio of prescription and non-prescription pharmaceuticals in urban and rural areas.

Classification		Prescription Pharmaceuticals	Non-Prescription Pharmaceuticals	Ratio
Total area	Concentration (µg/L)	0.0531	0.0947	1.8
	Detection frequency (%)	41.7	20.8	2.0
Urban area	Concentration (µg/L)	0.0727	0.0168	4.3
	Detection frequency (%)	56.3	29.7	1.9
Rural area	Concentration (µg/L)	0.0157	0.0222	1.4
	Detection frequency (%)	34.4	16.4	2.1

**Table 3:** Correlation between potential pollutant sources and concentrations of the pharmaceuticals in urban and rural areas.

Number of Livestock (Units)	Correlation	Land Use Area (km^2^)	Correlation	Usage ** and WWTPs	Correlation
Dairy cattle	0.742 *	Cropland	0.553 *	Total pharmaceutical usage	0.555
Swine	0.741 *	Paddy land	0.645 *	Total pharmaceutical usage except for ACE and NPX	0.948
Poultry	0.831 *	Forest land	0.663 *	Population not connected to WWTPs	0.316
Total of livestock	0.827 *	Total of land	0.649 *	Population not connected to WWTPs except for Seoul	0.760 *

* 95% confidential level (*p* < 0.05), ** Korea Pharmaceutical Manufacturers Association (KPMA).

## Data Availability

Not applicable.

## Supplementary Materials

The following are available online at https://www.mdpi.com/article/10.3390/ijerph18157969/s1, Table S1: Physicochemical properties of the target pharmaceuticals, Table S2: Information regarding WWTPs investigated in this study, Table S3: Analytical MS/MS conditions and retention time of the target pharmaceuticals and Table S4: Summary results for mean concentration and frequency detection of pharmaceuticals at each site of Han River watershed.
