# Supplementary material for: Spatio-Temporal Distribution and Influencing Factors of Human and Veterinary Pharmaceuticals in the Tributary Surface Waters of the Han River Watershed, South Korea
Source: Int J Environ Res Public Health. 2021 Jul 28;18(15):7969. doi: 10.3390/ijerph18157969 (PMC8345536; doi:10.3390/ijerph18157969)
Supplement: Supplementary file 1 [file ijerph-18-07969-s001.zip › ijerph-1306857-supplementary.pdf]

Supplementary Materials

Table and Figure of Contents

Table S1. Physicochemical properties of the target pharmaceuticals.

Table S2. Information regarding WWTPs investigated in this study

Table S3. Analytical MS/MS conditions and retention time of the target pharmaceuticals.

Table S4. Summary results for mean concentration and frequency detection of pharmaceuticals at each sites of Han River watershed.

Table S1. Physicochemical properties of target pharmaceuticals.

Compounds	Structure	Water solubility (mg/L)	pK_a	$LogK_{ow}$	$LogK_{oc}$	$LogD_{ow}$	Half-life surface water (hour)	Henry's law constant (atm m^3/mol)
Naproxen	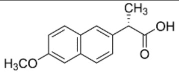	4.41×10^{1}	4.39	3.18	2.525	0.33	360	3.39×10^{-10}
Acetylsalicylic acid	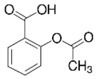	4.6×10^{3}	3.5	1.19	2.0	-2.31	151.2	1.30×10^{-9}
Carbamazepine	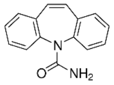	1.77×10^{1}	13.9	2.45	3.59	2.45	900	1.08×10^{-10}
Trimethoprim	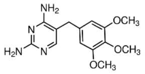	2.33×10^{3}	7.12	0.91	2.857	1.13	1440	2.39×10^{-14}
Clarithromycin	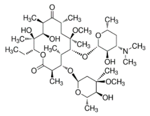	3.42×10^{-1}	8.99	3.16	2.174	2.31	-	1.73×10^{-29}
Sulfamethazine	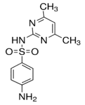	1.12×10^{4}	7.6/2.3	0.89	2.282	0.66	900	3.05×10^{-13}
Sulfamethoxazole	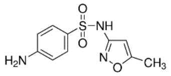	3.94×10^{3}	5.7/1.8	0.89	2.412	-0.43	900	9.56×10^{-13}
Sulfathiazole	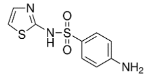	3.73×10^{2}	2.0/7.1	0.047	2.3	-0.22	-	5.80×10^{-14}

Water solubility, octanol-water partition coefficient (Log K_{ow}), soil organic carbon-water partition coefficient (Log K_{oc}), pK_a and distribution coefficients (Log D_{ow} at pH =7) for ionizable compounds and surface water half-life were compiled using EPI Suite.

Table S2. WWTPs information investigated in this study

WWTP (name)	Area	Capacity (m^2 d^{-1})[a]		Treatment technologies
		Design	Operation	
WWTP-1 (Wonju)	SHR	156,000	120,595	NPR
WWTP-2 (Moonmak)	SHR	7,000	4,811	BNR
WWTP-3 (Gwangju)	SHR	25,000	24,572	HBR, SBAF
WWTP-4 (Gonjiam)	SHR	23,000	21,694	SBF
WWTP-5 (Yangbeol)	SHR	20,000	7,216	SBAF
WWTP-6 (Samri)	SHR	5,000	4,211	SBAF
WWTP-7 (Icheon)	SHR	43,000	40,277	DeNiPho
WWTP-8 (Danweol)	SHR	2,000	1,863	MSBR
WWTP-9 (Yongmoon)	SHR	4,900	3,388	MBR
WWTP-10 (Danweol)	SHR	1,700	1,432	KNR
WWTP-11 (Injebukmeon)	NHR	2,000	1,482	SBR
WWTP-12 (Cheongsan)	IHR	2,100	1,125	MSBR
WWTP-13 (Dongducheon)	IHR	86,000	74,327	CNR
WWTP-14 (Guri)	HR	160,000	150,169	MLE
WWTP-15 (Jingeon)	HR	100,000	107,981	CSBR
WWTP-16 (Tancheon)	HR	900,000	733,833	MLE
WWTP-17 (Gwacheon)	HR	30,000	20,577	DeNiPho
WWTP-18 (Seongnam)	HR	460,000	346,611	CAS
WWTP-19 (Jongrang)	HR	1,590,000	1,234,376	CAS, MLE, SBAF
WWTP-20 (Parkdal)	HR	250,000	126,348	CSBR
WWTP-21 (Suksu)	HR	300,000	226,695	MLE
WWTP-22 (Gulpo)	HR	900,000	672,461	DeNiPho
WWTP-23 (Geumchon)	HR	44,000	28,853	MBR, HDF
WWTP-24 (Samsong)	HR	16,000	7,153	CSBR

[a]average daily value (2015), NPR: nitrogen and phosphorus removal process, BNR: biological nutrient removal process, SBAF: SK biological aerated filters, SBF: suspended bio filter, HBR: suspended bio filter, MSBR: modified sequencing batch reactor, DeNiPho: activated sludge treatment with biological removal of nitrogen and phosphorous, KNR: Kwon's nutrient removal, MBR: membrane bio reactor, SBR: sequencing batch reactor, CNR: cilium nutrient removal process, MLE: modified Ludzack-Ettinger process, CSBR: constants sequencing batch process, CAS: conventional activated sludge process, HDF: Hanhwa dynamic flow

Source: South Korea Ministry of Environment, 2016-17 Sewerage statistics and 2016-17 Operation status of wastewater treatment plants.

Table S3. Analytical MS/MS conditions and retention time of the target pharmaceuticals.

Compounds	R.T.	Precursor (m/z)	Quantitation ion (m/z)	Cone energy (V)	Collision energy (V)	Ionization mode
ASA	4.07	137.1	46.1	10	9	ESI(-)
NPX	7.52	229.1	170	18	15	ESI(-)
CBZ	6.02	237.2	194.2	36	18	ESI(+)
CTM	5.9	748.3	158.1	38	28	ESI(+)
TMP	2.5	291.1	230.2	40	22	ESI(+)
SMX	4.33	253.9	156	28	17	ESI(+)
SMZ	3.39	279.1	186	32	17	ESI(+)
STZ	2.62	255.9	156	22	17	ESI(+)

Table S4. Summary results for mean concentration and frequency detection of pharmaceuticals at each site of Han River watershed.

Sites	Mean Concentration ($\mu g\ L^{-1}$)	Frequency detection (%)
SR-1	0.0841	37.5
SR-2	0.0442	34.4
SR-3	0.0522	34.4
SR-4	0.0678	65.6
SR-5	0.0201	31.3
SR-6	0.0071	6.3
SR-7	0.0107	15.6
NR-1	0.0857	6.3
NR-2	0.0345	12.5
NR-3	0.0131	21.9
NR-4	0.0095	25.0
NR-5	0.0223	12.5
IHR-1	0.1591	75.0
IHR-2	0.0189	46.9
IHR-3	0.0632	3.1
IHR-4	0.0234	50.0
HR-1	0.1087	59.4
HR-2	0.1530	50.0
HR-3	0.0226	37.5
HR-4	0.2281	53.1
HR-5	0.1874	53.1
HR-6	0.0315	31.3
HR-7	0.1042	59.4
HR-8	0.0526	53.1
